# Supplementary figures and images for: Dissection of the Genetic Basis of Resistance to Stem Rot in Cultivated Peanuts (Arachis hypogaea L.) through Genome-Wide Association Study
Source: Genes (Basel). 2023 Jul 14;14(7):1447. doi: 10.3390/genes14071447 (PMC10378806; doi:10.3390/genes14071447)

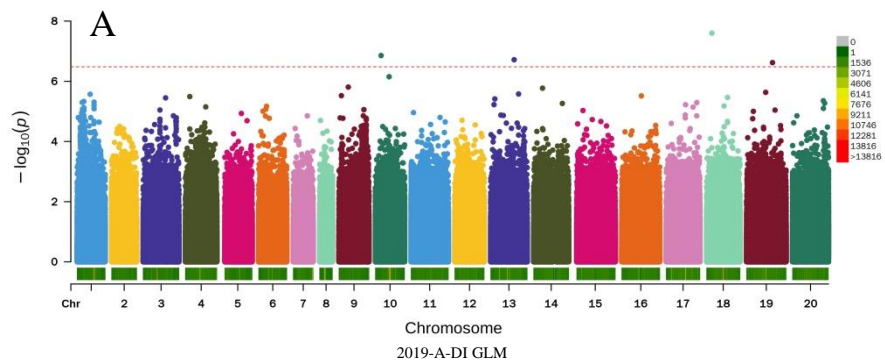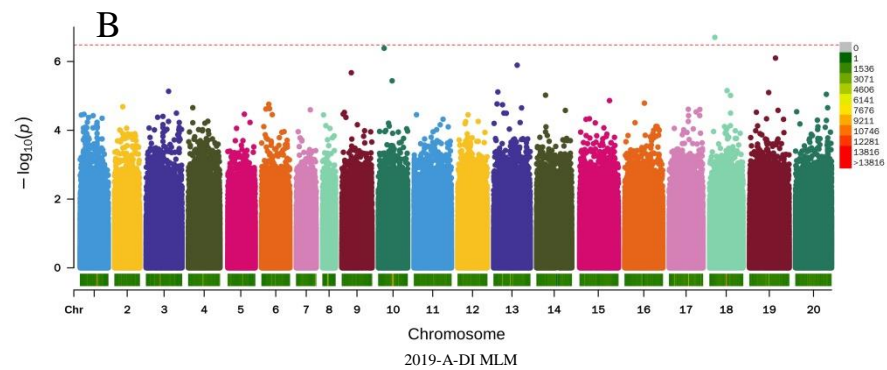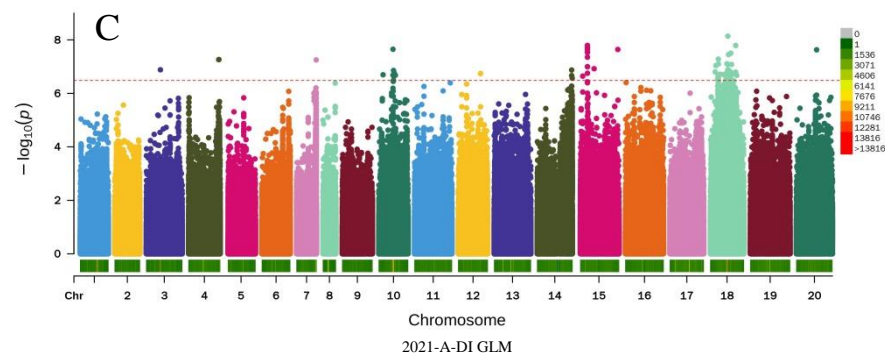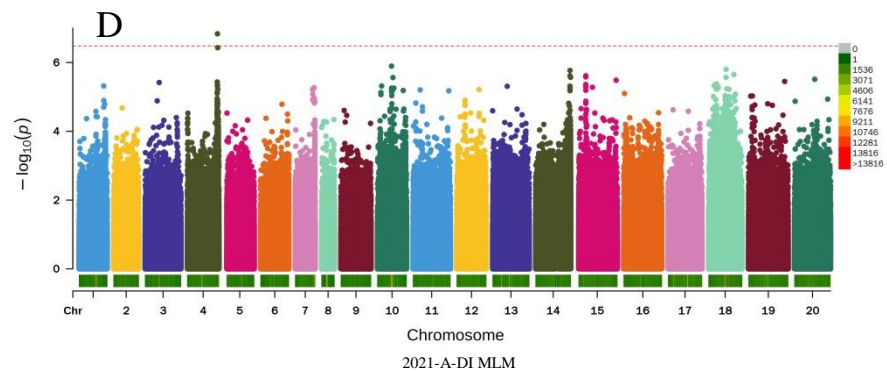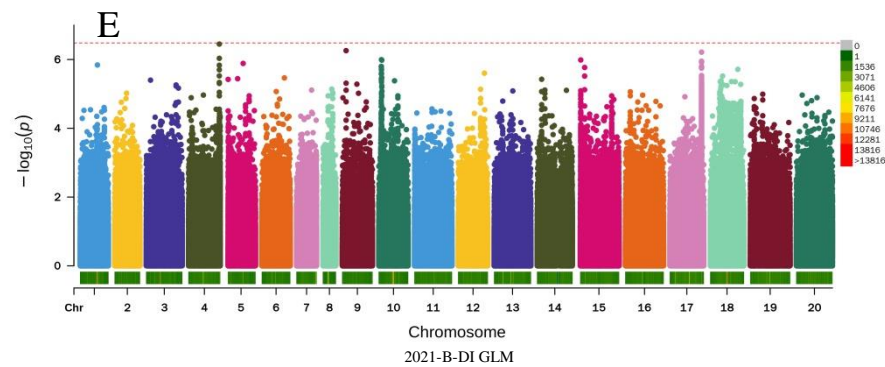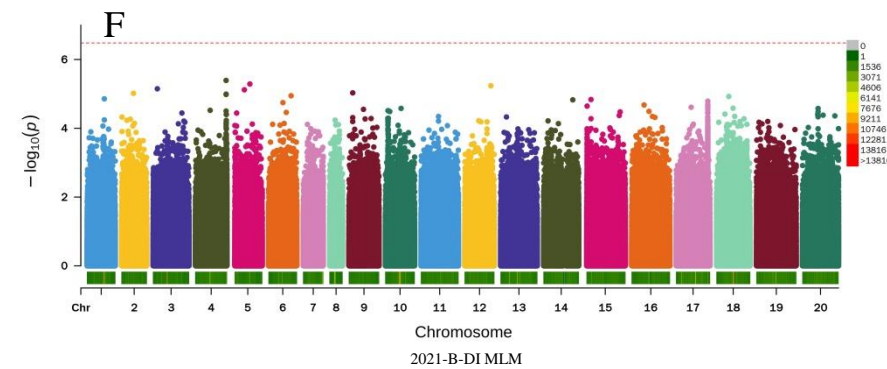

Supplement: Supplementary file 1 [file genes-14-01447-s001.zip › Fig S1 Mahattan plots produced by GLM and MLM models across three enviroments.pdf]
